# The Influence of Gender Inequality in the Development of Job Insecurity: Differences Between Women and Men

**DOI:** 10.3389/fpubh.2020.526162

**Published:** 2020-10-09

**Authors:** Sara Menéndez-Espina, Jose Antonio Llosa, Esteban Agulló-Tomás, Julio Rodríguez-Suárez, Rosana Sáiz-Villar, Héctor Félix Lasheras-Díez, Hans De Witte, Joan Boada-Grau

**Affiliations:** ^1^Department of Psychology, Oviedo University, Oviedo, Spain; ^2^Department of Health Sciences, Universidad Internacional de La Rioja (UNIR), Logroño, Spain; ^3^Research Unit Occupational & Organizational Psychology and Professional Learning (O2L) KU Leuven, Leuven, Belgium; ^4^Optentia Research Focus Area, North-West University, Vanderbijlpark, South Africa; ^5^Department of Psychology, Rovira i Virgili University, Tarragona, Spain

**Keywords:** job insecurity, temporary work, gender perspective, non-standard work, occupational health

## Abstract

Job insecurity is an indicator of precarious work that refers to the fear of losing one's job. It is a relevant source of stress, with negative consequences on people's mental health. The main objective and contribution of this study is to identify how gender inequality and job insecurity are related, responding to the lack of consensus found in scientific literature in this field of study. To do so, a predictive study of job insecurity, broken down by gender, is developed, considering sociodemographic and labor variables as antecedents. The sample included 1,005 employees (420 men and 585 women) aged between 18 and 65, and a linear regression was conducted for each group. Results show that women perceive greater insecurity under precarious working conditions (temporary work, informal work, salary cuts, tenure), whereas in the case of men variables related to their professional careers (job category, education) and household incomes were relevant predictors. It is concluded that job insecurity affects both gender groups, but the conditions in which this perception grows are significantly impacted by gender inequality. These findings will allow for holistic and effective actions to decrease the effects of precarious work.

## Introduction

Perceived job insecurity is understood, in its original formulation, as “the perceived powerlessness to maintain the desired continuity in a threatened job situation” (1). It is also defined as “one's expectations about continuity in a job situation” (2) or “the general concern of employees in terms of the future continuity in a desired job situation” (3). It is a field of study resulting from research into work-related stress and its harmful impact on health, at a time when some of the dimensions of the welfare state are being questioned. The origin of the study of job insecurity coincides with the 1973 oil crisis and the expansion of liberal policies fostered by Margaret Thatcher and the other conservative governments during the 1980s. From then on, globalization is consolidated giving way to what Bauman (4) describes as “liquid employment,” where a decline of labor and social rights of the Keynesian welfare system occurred. This system emerged as a social pact after World War II and was based on the possibility of a full employment paradigm, where access to labor market implied access to the system of social protections. The result of this change is a type of weak, flexible and uprooted (5) and, ultimately, precarious (6, 7) employment. This evolution that summarizes the trend over the last quarter of the twentieth century is aggravated during the global economic crisis at the turn of the new millennium. Beck (8) states that, in a framework in which employment tends toward a progressive deregulation of rules, there is greater interest in analyzing precarious conditions by studying the perceptions of employees and not only through the formal features of labor relations. Thus, over recent years there has been increasingly more interest in studying job insecurity (9). Current precarious labor relations and conditions are based on flexibility and instability, and perceived job insecurity is understood, in this article, as a highly relevant tool to assess the effects and experience of flexible employment (10). In other words, it connects the social reality of work and the personal experience of that reality. Therefore, job insecurity is a subjective (1), involuntary and uncontrollable (11) process that anticipates the loss (2) of a labor situation that one wishes to maintain (3).

The avenues of research on perceived job insecurity consider this phenomenon as a stressor, assuming that flexible and deregulated labor relations and conditions always imply some discontent. Thus, subjective job insecurity has been associated with negative and dysfunctional effects on employees' well-being (12). Specifically, it has been observed effects on general mental health (13), depression (14), anxiety (15), and even in relation to suicidal ideation (16), as well as to physical well-being (17) or specific syndromes related to physical health, as for example heart conditions (18, 19). The study of job insecurity has a long history in Anglo-Saxon contexts, and it is still a highly relevant and current topic (20). Job insecurity is a dynamic construct in terms of its development, related to the economic and social circumstances of the country or the region subject to study. Thus, Lübke and Erlinghagen (21) show the differences in perceived job insecurity in the different countries, according to the situation of the labor market in the past, the current economic situation and the welfare policies implemented. Likewise, recent literature reviews have confirmed this fact, finding that as unemployment rates grow, there are higher levels of job insecurity (22, 23).

In addition to the socioeconomic context that justifies and urges further research on job insecurity, some aspects of this phenomenon still must be identified, and others have to be studied in greater depth. One of them refers to the situation of women. More specifically, the way in which gender inequality, historically generated in the employment scenario (24, 25) is related to job insecurity. There is extensive empirical evidence showing differences between men's and women's work based on discrimination against women (26–29), which are repeated and increase over the years (25, 30, 31). This fact is translated in specific phenomena such as the gender pay gap, occupational segregation, higher rates of part-time and informal employment in women, as well as a greater burden of care work (25, 27). However, we believe that it is also necessary to know its implication in the characteristics and development of job insecurity.

The study of different aspects of job insecurity had already been presented previously from a gender perspective (32–36). However, there is no consensus in the scientific literature regarding the relationship established between job insecurity and gender. It is difficult to answer the question of which group scores higher in this variable, and which is more affected by the consequences. Traditionally, this experience was considered to occur to a greater extent among men (37). This conception responds to the model of man as the breadwinner (38). In households where the woman is the main breadwinner, no differences with men are found (39). Other studies, in contrast, have found a greater degree of job insecurity among women (12, 40, 41). These results are to be expected because women usually undertake more flexible, temporary and part-time jobs (25, 29, 42). A third view, wider spread and embraced, considers that there are no gender differences in relation to job insecurity, understanding this as a source of stress both in men and in women (36, 43, 44). Likewise, Gaunt and Benjamin (33) find that the differences derive from whether women with paid jobs have a more traditional or household work-oriented ideology, or on the contrary a more egalitarian ideology, namely, more focused on their professional careers. Thus, women belonging to the first group suffer less job insecurity than women in the second group. Anyhow, the trend is to focus these studies on male-female couple households, although today there is a wider variety of household models. Keim et al. (22) take a more global approach, establishing a relationship between increased job insecurity scores in men and women and the rise of unemployment rates in the country of residence, as is the case of Spain. Thus, the scenario of the study on job insecurity in men and women is characterized by a great disparity of results, confirming the need to design research studies based and developed in agreement with the criteria and recommendations of the gender perspective (45). Social inequality between men and women occurs in all life domains, but it is in employment where it is more obvious and concerning (25). In this context, we think that job insecurity must necessarily be a phenomenon that is affected by this inequality. Therefore, this research proposes a new approach to study gender differences in job insecurity: we want to test that there are no differences between men and women as a general measure, but they exist when we analyse the objective background that facilitates its appearance.

H1: There is no difference between the general score for job insecurity in Men and women

Several studies have looked into predictors or precursors of job insecurity, including gender (9, 22, 46–48). Out of all classifications of this type of predictive variables, our benchmark has been Probst and Lavaysse (23). We chose two types of factors: first, demographic variables, and, second, variables related to labor conditions. Both are under the umbrella of what these authors call individual and objective factors. We understand that these individual and objective factors place certain population groups in a weaker labor position, facilitating the loss of employment. Previous research has found a greater perception of job insecurity (49). Urbanaviciute et al. (50) refer to this weak labor position as a precariousness profile.

The demographic set includes variables such as age or academic education, as well as certain household characteristics like having children and household income. In terms of age, studies found that job insecurity increases with the passing of time (48). However, other authors found no linear relationship between both variables (51). Fullerton and Wallace (52) even describe a curvilinear relationship. This means that the youngest and oldest employees have less fear of losing their jobs. However, studies conducted after the 2008 economic crisis seem to refute this idea. According to Keim et al. (22), young people experience most insecurity, and this relationship is aggravated at times with higher unemployment rates. In the same line, Buonocore et al. (26) have identified specific generational differences: people born between 1980 and 1994 tended to perceive higher levels of job insecurity than previous generational groups at the time of the study, carried out in 2010. In terms of education, job insecurity has traditionally been related to a lower educational level (53), although more recent studies show an inverted relationship (54, 55). Thus, Kinnunen et al. (56) and Keim et al. (22) state that it also depends on macroeconomic variables, so that when there are high unemployment rates, people with higher qualifications have the highest job insecurity levels. In contrast, when the job offer is sufficient, having higher education levels dampens job insecurity. On the other hand, household-related variables have also been analyzed as possible predictors of perceived job insecurity. The studies by Näswall and De Witte (48) and Richteret al. (35) compare the impact of having dependent children in different countries, without concluding that it is a predictor of job insecurity. A similar study by Muñoz de Bustillo and de Pedraza (47), only establishes a significant positive relationship between having dependent children and the development of subjective job insecurity in the case of some European Mediterranean culture regions. Another variable of interest is the family's economic situation, related to the main breadwinner (35, 36), and the existence of a spouse who also works and contributes to the household income. In this respect, Mauno and Kinnunen (57) observe, in man–woman couples with two salaries, that the concern for the economic situation of one of the spouses generates greater job insecurity in the other one.

*H2: Job insecurity increases in a more vulnerable socio-economic context, such as (a) lower household income, (b) having children, and (c) a lower level of education*.

The second set includes predictive variables related to labor conditions. Keim et al. (22) and Shoss (9) have identified these factors. Temporary and part-time contracts increase job insecurity (48, 58, 59). Moreover, a longer time spent in a company is associated with less job insecurity (21). The same applies to professional categories: white collar workers or with a higher qualifications perceive less insecurity than blue collar workers (60). Changes in the organization, such as reorganizations or salary restructuring, salary cuts or staff reductions also increase insecurity (9, 23). Other type of employees that is highly susceptible to experience job insecurity are self-employed workers (61). We believe that they require more in-depth studies. This was also highlighted by Shoss (9), given the increase in self-employed workers in recent years (e.g., Eurostat 2017 data). In addition, informal work, without contractual relationships, is presented as an important form of precariousness with serious implications for insecurity (62) and health (63).

*H3: The level of job insecurity increases in the case of being in a weak Labor position, such as (a) informal work, (b) temporary contracts, (c) part-time work and (d) having suffered a salary cut in the last year*.

*H4: In a stronger Labor position, such as (a) a longer tenure and (b) a white-collar job category, the job insecurity decreases*.

## This Study

In this study we incorporate social and contextual factors as precursors of job insecurity instead of intrapsychic variables. The reason is that these factors do not consider the individual to be the origin of the insecurity, but rather the surrounding social circumstances. We want to provide evidence on the weight that this type of variables has on the perception of job insecurity. To do so, our first objective is to analyse different socioeconomic factors that affect to job insecurity. A second objective is to incorporate the gender perspective to previous knowledge in this area. This allows us to propose different levels of organizational and institutional interventions, showing that we are facing a psychosocial phenomenon that affects workers. So, the research question asked is: do the demographic and labor factors related to job insecurity have a different effect on women and men? This question is based on the weaker position that women typically occupy in the labor market, along with other factors that are also related to greater difficulty in accessing employment. More recent scientific literature concludes that job insecurity is experienced equally by men and women, although there is no clear consensus. The reasons for this could be the limited number of gender-based studies on the topic that consider the unequal conditions of female work.

## Materials and Methods

### Participants

Quota and convenience sampling were conducted. To this end, the target group for the study was defined as men and women between 18 and 65 years of age, who were working and resided in Spain. Then, volunteers were sought to complete the questionnaire online during a period of 6 months. Once we had a sufficient number of participants, the well-completed questionnaires and those that answered the requested profile were filtered. The sample obtained comprises 1,005 individuals, living in different Spanish regions: 420 men and 585 women, with an average age of 36.03 years (SD = 12.24). The participants' characteristics are summed up in Table 1.

**Table 1:** Means, standard deviations and frequencies of the socio-economic and labor variables.

**Variables**	**Total**	**Men**	**Women**
Age	36.03 (12.24)	36.66 (13.21)	35.57 (11.49)
Household income^*^ (Euros/month)	2120.68 (1296.35)	2404.19 (1449.35)	1918.47 (1133.95)
Number of children	0.38 (0.69)	0.31 (0.65)	0.42 (0.71)
Tenure (months)	104.48 (146.46)	119.6 (150.79)	97.77 (142.06)
Education			
University education = 0	332 (33%)	169 (50.9%)	163 (49.1%)
No university education = 1	673 (67%)	251 (37.3%)	422 (62.7%)
Job category			
White collar = 0	198 (19.7%)	100 (50.5%)	98 (49.5%)
Blue collar = 1	807 (80.3%)	320 (39.7%)	487 (60.3%)
Temporary work			
Temporary contract = 1	568 (56.5%)	207 (36.4%)	361 (63.6%)
Open-ended contract = 0	437 (43.5%)	213 (48.7%)	224 (51.3%)
Part-time Work			
Part-time day = 1	270 (26.9%)	93 (34.4%)	177 (65.6%)
Full day = 0	735 (73.1%)	327 (44.5%)	408 (55.5%)
Informal work			
With contract = 0	897 (89.3%)	380 (42.4%)	517 (57.6%)
Without contract = 1	108 (10.7%)	40 (37%)	68 (63%)
Self-employment			
Self-employed = 1	101 (10%)	55 (54.5%)	46 (45.5%)
Employee = 0	904 (90%)	365 (40.4%)	539 (59.6%)
Salary cut			
Cut in last year = 1	199 (19.8%)	73 (36.7%)	126 (63.3%)
No cut in last year = 0	780 (77.6%)	338 (43.3%)	442 (56.7%)

Standard deviation and percentages on brackets. N (men) = 420; N (women) = 585;

* *net income; Confidence interval: 95%*.

### Instruments

Version JIS-8 of the Job Insecurity Scale (64), validated in the Spanish population by Llosa et al. (10) has been used to measure Job Insecurity. The original scale shows a realiability above 0.80 with the Conbrach's alpha test; in the Spanish validation a index of α = 0.90 was obtained. The scale comprises 8 items on a 5-point Likert scale format. It offers a global score on Job Insecurity, as well as for two dimensions: cognitive and affective. The range of the sum scores is from 8 to 40.

The coding of socio-demographic variables and working conditions-related variables have been taken from European surveys drawn up by Eurofound, such as the European Working Conditions Survey (EWCS) 2010 version, European Social Survey (ESS), version number 6, and the International Standard Classification of Occupations (ISCO-08). The Age, Household Income and Tenure variables are numerical. The other variables have been transformed into dichotomic variables or dummy variables (65), and take the following values: informal work (0 with contract, 1 without contract); Temporary Work (1 temporary contract, 0 open-ended contract), Part-time work (1 part-time day, 0 full day); Job Category (0 white collar, 1 blue collar); Self-employment (1 self-employed worker, 0 third-party worker), and Education (0 with university studies, 1 without university studies). We also added a variable related to changes in the company, which is Salary Cut (1 salary cuts in last year, 0 no cuts in salary in last year).

### Procedure

The respondents completed a self-administered questionnaire with all variables. All participants were informed of the objectives, characteristics, and procedures of the study, and signed the informed consent prior to completing the questionnaire. This research follows the requirements and protocols of the Ethics Committee of Oviedo University, where it was performed, as well as all the ethical demands and recommendations included in section 8 of the Ethical Principles of Psychologists and Code of Conduct of the APA (66).

### Data Analysis

First, a descriptive analysis of the data was conducted to determine the sample characteristics and the average scores on the JIS-8 scale. Then, a Two-Sample *T*-Test was carried out to compare the score in job insecurity between women and men.

A stepwise linear regression analysis was conducted separately for men and women. Socio-economic type variables (Age, Education, Household Income, and Number of Children) were used, as well as labor type variables (Informal Work, Temporary Work, Part-time Work, Tenure, Job Category, Salary Cut and Self-employment). A *P*-value of 0.05 was chosen as the significance level in the analysis.

## Results

The results of the Two-Sample *T*-Test are displayed in Table 2. In general, women show a higher average score in Job Insecurity (M = 21.9; SD = 6.94) than men (M = 19.5; SD = 7.17; *t* = −5.420; *P* < 0.001).

**Table 2:** Two-Sample *t*-Test for Job Insecurity in women and men.

		**Levene test**		***t*****-test**		
**Means (SD)**		**F**	**P**	***t***	**df**	***P***
Men	19.5 (7.17)	0.635	0.426	−5.420	1,003	0.000
Women	21.9 (6.94)					

Table 3 includes the correlations between the dependent variable (Job Insecurity) and the numerical predictors, both for men and for women. In males, Job Insecurity statistically significantly and positively correlates with Education (*r* = 0.115), Temporary Work (*r* = 0.329), and Salary Cut (*r* = 0.234); it does so negatively with Household Income (*r* = −0.326), Number of Children (*r* = −0.140), Job Category (*r* = −0.189), Tenure (*r* = −0.258), and Self-employment (*r* = −0.141). In women, Job Insecurity statistically significantly and positively correlates with Temporary Work (*r* = 0.363), Informal Work (*r* = 0.097), Job Category (*r* = 0 −0.124), and Salary Cut (*r* = 0.193); it does so negatively with Household Income (*r* = −0.181), Tenure (*r* = −0.297), and Self-employment (*r* = −0.087). In general, all the variables correlate in the same direction with Job Insecurity in men and women, except Education, which does so positively in men and negatively in women. Job Insecurity and Age do not correlate in any of the groups. Therefore, the variable Age was not included in the regression analysis.

**Table 3:** Correlations between Job Insecurity and the socio-economic and labor variables in men and women.

	**1**.	**2**.	**3**.	**4**.	**5**.	**6**.	**7**.	**8**.	**9**.	**10**.	**11**.	**12**.
1.Job insecurity	1	−0.089	−0.326^**^	−0.140^**^	0.115^*^	−0.258^**^	0.329^**^	0.086	0.052	−0.189^**^	−0.141^**^	0.234^**^
2.Age	−0.017	1	0.070	0.377^**^	0.005	0.714^**^	−0.432^**^	0.016	−0.307^**^	0.061	0.142^**^	0.110^*^
3.Income	−0.181^**^	−0.008	1	0.065	0.112^*^	0.194^**^	−0.149^**^	−0.014	−0.057	0.111^*^	0.146^**^	−0.058
4.N. of children	−0.018	0.318^**^	−0.047	1	0.027	0.253^**^	−0.243^**^	−0.019	−0.139^**^	0.002	0.148^**^	0.055
5.Education	−0.020	−0.023	0.165^**^	−0.022	1	0.030	0.061	0.048	−0.042	0.123^*^	−0.070	0.002
6.Tenure	−0.297^**^	−0.580^**^	0.187^**^	0.152^**^	0.128^**^	1	−0.451^**^	−0.047	−0.295^**^	0.092	0.093	0.058
7.Temporary W.	0.363^**^	−0.345^**^	−0.165^**^	−0.130^**^	−0.129^**^	−0.486^**^	1	−0.329^**^	0.231^**^	−0.142^**^	0.013	0.070
8.Informal work	0.097^*^	0.075	−0.039	0.087^*^	0.120^*^	−0.022	−0.286^**^	1	−0.100^*^	0.009	−0.379^**^	−0.105^*^
9.Part-time W.	0.075	−0.247^**^	−0.053	−0.155^**^	−0.072	−0.236^**^	0.228^**^	−0.191^**^	1	−0.025	−0.037	−0.002
10.Job category	−0.124^**^	−0.159^**^	0.179^**^	−0.094^*^	0.150^**^	0.122^**^	−0.165^**^	0.109^**^	−0.163^**^	1	0.101^*^	−0.050
11.Self-emp.	−0.087^*^	0.176^**^	0.046	0.014	−0.003	0.170^**^	−0.031	−0.310^**^	−0.082^*^	0.063	1	0.018
12.Salary cut	0.193^**^	0.115^**^	−0.134^**^	0.074	−0.085^*^	−0.045	0.115^**^	−0.021	0.093^*^	−0.110^**^	0.106^*^	1

Men above the diagonal; Women below the diagonal;

* P < 0.05;

** *P < 0.01; Confidence interval: 95%*.

The results of the linear regression can be seen in Table 4. For the sample of men, the model obtained includes the following predictor variables, in order of importance in the model: Temporary Work (β = 0.314), Household Income (β = −0.258), Salary Cut (β = 0.263), Informal Work (β = 0.204), Education (β = 0.132), and Job Category (β = −0.137). For the sample of women, the resulting model contains the variables, Salary cut (β = 0.119), Temporary Work (β = 0.361), Informal Work (β = 0.181), and Tenure (β = −0.293). Number of Children, Part-time work, and Self-employment were not statistically significant variables in any of the samples, so they do not predictor Job Insecurity. The model explains 28.7% of variance in men and 23% in women.

**Table 4:** Results of multiple linear regression analysis of predictors associated with job insecurity in men and women (standardized coefficients).

	**Men**		**Women**	
**Predictors**	**β**	**ΔR^**2**^**	**β**	**Δ*R*^**2**^**
Temporary work	0.314^**^	0.108^**^	0.361^**^	0.132^**^
Informal work	0.204^**^	0.43^**^	0.181^**^	0.044^**^
Salary cut	0.220^**^	0.039^**^	0.119^**^	0.022^**^
Househols income	−0.263^**^	0.078^**^	-	-
Education	0.132^**^	0.017^**^	-	-
Job category	−137^**^	0.014^**^	-	-
Tenure	-	-	−0.239^**^	0.010^*^
Number of children	-	-	-	-
Part-time work	-	-	-	-
Self-employment	-	-	-	-
*R*^2^ adjusted	0.287^**^		0.230^**^	

β, Standardized coefficient; R^2^ adjusted, Adjusted percentage of variance explained; ΔR^2^, Change in percentage of variance explained;

* P < 0.05;

** *P < 0.01; Confidence interval: 95%*.

## Discussion

The objective of this study is to determine the existing gender differences when predicting perceived job insecurity based on individual and organizational objective variables. Previous studies took gender as a job insecurity predictor (22, 37, 48) but this article, compared the results in men and women to study the influence of the social and cultural context on job insecurity. Generally speaking, it has been found that differences in men and women with respect to the development of job insecurity reflect gender inequality at work. Women showed a higher score in job insecurity than men, taking a general measure and without controlling the influence of other variables. So, Hypothesis 1 (There is no difference between the general score for job insecurity in men and women) was not confirmed. Regarding Hypothesis 2, (Job insecurity increases in a more vulnerable socio-economic context, such as (a) lower household income, (b) having children, and (c) a lower level of education) was not satisfied, as a higher level of Job Insecurity was observed in men workers with university studies. Finally, Hypotheses 3 (The level of job insecurity increases in the case of being in a weak labor position, such as (a) informal work, (b) temporary contracts, (c) part-time work, and (d) having suffered a change in the contract in the last year), and Hypothesis 4 (H4: In a stronger labor position, such as (a) a longer tenure and (b) a white collar job category, the job insecurity decreases) were corroborated, but differently in men and women, because there are some elements that appear in both genders, but others only in one of the groups.

If we look at the common variables, we find some related to working conditions. The results obtained point to temporary work, informal work and salary cuts in the last year as predictors in both groups. These are variables associated with a weak labor market position (67) that had previously been defined as predictors of job insecurity (9). The fact that organizational changes generate greater fear of losing one's job becomes especially important (22, 68), as having experienced a cut in salary in the last year.

If we consider the specific conditions or variables that present differences between the models obtained, we find the following: household income level, education and job category are statistically significant in the group of men, but not so in the group of women. The opposite occurs with tenure, which, in our results, only appears in the female sample model. Regarding income, the discrepancies in this variable are explained by maintaining the model that understands the man as the main breadwinner (Lewis, 2001), since, when the men belong to a household with low income, the fear of losing their job increases. Thus, although both men and women work in the family unit, the female salary is still assumed today as secondary and complementary to the male partner, as Giesselman (69) observed in European countries such as Germany. The results referring to the influence of education reflect a changing reality: they show that job insecurity also grows at higher educational and professional levels in the men sample. This phenomenon is being observed among general youth (54, 55), and may be playing an important role in unfulfilled labor expectations. Its relevance in the group of men and not in that of women could be related to their tendency to focus more on their professional careers, while women are forced to divide their attention between work and family (42). Moreover, this is one of the factors that generates occupational segregation, especially the so-called glass ceiling (27). The relation is inverse with the job category, increasing the job insecurity among the blue collar workers, in the line of previous studies (9).

On the other hand, despite seniority in the company being related to insecurity, in this study we can observe that the threat of the loss is more relevant in women when they have not held the job for a long time. A possible explanation would be the expectations related to the presence of higher figures of precariousness for female work (25, 42), which could be increasing women's fear of being fired or not having their contracts renewed. Authors such as Lübke and Erlinghagen (21) sustained that greater seniority in the company decreases job insecurity, and our results extend this idea with the gender perspective. However, seniority in the company is a variable that is highly related to age and the identity that the job grants to people (46, 70, 71). Regarding age, the results do not support the existence of a linear relationship between age and job insecurity in any group, as Glavin and Young (51) found. Even so, studies that focus on specific age groups, such as youth or over-45 s, would be necessary in the job insecurity field of study. We must emphasize that labor relations in the framework of neoliberalism do not follow the linear path characteristic of Fordism (5, 72). Therefore, the variables age and seniority at the company do not maintain the same relationship with job insecurity.

In short, it is observed that the variables of success and professional career (job category, household income), maintained and reproduced by traditional roles, become more important in men. In contrast, women are conditioned by the highly discriminatory and segregating labor environment that often submits them to higher percentages of working poverty, part-time work, discrimination, lower salaries, etc. (25). These circumstances determine the expectations of both genders, so perceived job insecurity also varies. This goes to show and reflects that real equality at work has still not been achieved; but outside it has not been either. Women's lives are conditioned by their dual presence in two type of jobs: both in paid jobs and in the work required to care for and maintain the household (25, 27). The need for an in-depth social change regarding gender roles continues to exist, that will equal the conditions of men and women both in the labor environment and in life courses. Meanwhile, parity and equilibrium measures of these inequality contexts help to take further steps in that direction, such as the implementation of efficient equality, non-discrimination and conciliation policies for both genders (73).

## Practical Implications

These results were obtained in the context of the Spanish labor market, which is defined by a flexible neoliberal model, and with unemployment, temporary and poor worker rates above the European average, especially in women (55, 72). This complements other research on countries with similar labor models and provides knowledge about the effects of job insecurity in these contexts. Otherwise, the research show the need to conduct more gender-sensitive studies. It is a strategy that does more than just include the variable in the analysis, as it requires considering the background, relations and consequences that a social and cultural context of gender inequality has on the phenomenon studied. In this way it will be possible to understand questions not solved in scientific literature or increase knowledge to propose and reach more efficient solutions that adapt to today's social reality. In agreement with the results obtained, it is important to maintain this vision, as well as to resume and copy studies conducted by other authors, bearing in mind the influence of gender.

On the other hand, the psychological approach used to address topics such as the one we present here also has implications. In our study, the gender differences found in the development of job insecurity were proposed from a psychosocial approach, not giving priority to intrapsychic variables. The results support the fact that anticipating the loss of a job is not only explained by this type of individual factors, and that the variables relating to the environment are also relevant (46). Thus, they show that today's labor context, marked by instability, leads people to develop a perception of insecurity, placing its origin at levels above individual psychological differences. This means that the responsibility for the appearance of discontent associated with work does not correspond to the individual, but to the precarious conditions. Otherwise, this is a phenomenon that cannot only be tackled by interventions with the workers, but rather, a holistic approach is required that considers both the individual level (repairing), and the social level (organization and contextual). With all of this, and given the deterioration that job insecurity generates in workers (12, 13, 74), its prevention must be a necessary and urgent question. Resuming the Keynesian work model is not possible, but putting political, legal, economic initiatives, etc. into motion are essential, to guarantee greater stability in job careers, especially in women. Its objective would be to compensate the environment of flexibility and mobility that characterizes the current labor market and that favors discontent in workers.

## Limitations and Future Research

This research, however, presents certain limitations. There are three analysis variables that have not been significant in the model, contrary to what is expected: part-time work, number of children and self-employment. Working part time has been defined as a predictor of job insecurity by other authors (9). In the future, a more complete measure of this variable is recommended, especially due to its high presence in female paid work (29). Regarding having children and the number of them, Näswall and De Witte (48) and Richter, Näswall and Sverke (35) did not find any relationship with job insecurity, neither. Even so, the possibility of analyzing whether this variable is relevant faced with the fear of losing one's job in other more specific population profiles, such as women with different family and socioeconomic situations, for example, single mothers, is put forward. Finally, self-employed work has been introduced, not having considered it as a predictor of job insecurity in previous studies. Even so, it is necessary to develop job insecurity studies in self-employed workers, a vulnerable group insofar as social and economic conditions are concerned (61, 75). We also recommend delving into the new relationships observed between job insecurity and educational level, especially among young people, and with generational studies.

## Conclusions

The main contribution of this study with respect to knowledge about job insecurity refers to the differences found in men and women. In general, this perception appears under clearly differentiated conditions that respond to gender inequality at work (25, 42). In conclusion, although scientific literature determines that job insecurity is developed in the same way in men and women, in quantitative terms, differences of a different nature have been found that lead to a more in-depth knowledge of the background of this phenomenon and its implications.

## Data Availability Statement

The datasets generated for this study are available on request to the corresponding author.

## Ethics Statement

The studies involving human participants were reviewed and approved by Ethics Committee of Oviedo University. The patients/participants provided their written informed consent to participate in this study.

## Author Contributions

SM-E, JL, and EA-T: conception and design of the work. HL-D and RS-V: data collection. SM-E, JL, and JB-G: data analysis and interpretation. SM, JL, and JR-S: drafting the article. HD, EA-T, and JR-S: critical revision of the manuscript. All authors read and approved the final version of the manuscript.

## Conflict of Interest

The authors declare that the research was conducted in the absence of any commercial or financial relationships that could be construed as a potential conflict of interest.
